# Identification of critical residues of the serotype modifying *O*-acetyltransferase of *Shigella flexneri*

**DOI:** 10.1186/1471-2091-13-13

**Published:** 2012-07-15

**Authors:** Farzaana Thanweer, Naresh K Verma

**Affiliations:** 1 Division of Biomedical Science and Biochemistry, Research School of Biology, Australian National University, Canberra, ACT, 0200, Australia

**Keywords:** Shigella flexneri, *O*-acetyltransferase (Oac), Critical amino acids

## Abstract

**Background:**

Thirteen serotypes of *Shigella flexneri* (*S. flexneri)* have been recognised, all of which are capable of causing bacillary dysentery or shigellosis. With the emergence of the newer *S. flexneri* serotypes, the development of an effective vaccine has only become more challenging. One of the factors responsible for the generation of serotype diversity is an LPS O-antigen modifying, integral membrane protein known as *O*-acetyltransferase or Oac. Oac functions by adding an acetyl group to a specific O-antigen sugar, thus changing the antigenic signature of the parent *S. flexneri* strain. Oac is a membrane protein, consisting of hydrophobic and hydrophilic components. Oac bears homology to several known and predicted acetyltransferases with most homology existing in the N-terminal transmembrane (TM) regions.

**Results:**

In this study, the conserved motifs in the TM regions and in hydrophilic loops of *S. flexneri* Oac were targeted for mutagenesis with the aim of identifying the amino acid residues essential for the function of Oac. We previously identified three critical arginines–R73, R75 and R76 in the cytoplasmic loop 3 of Oac. Re-establishing that these arginines are critical, in this study we suggest a catalytic role for R73 and a structural role for R75 and R76 in *O-*acetylation. Serine-glycine motifs (SG 52–53, GS 138–139 and SYG 274–276), phenylalanine-proline motifs (FP 78–79 and FPV 282–84) and a tryptophan-threonine motif (WT141-142) found in TM segments and residues RK 110–111, GR 269–270 and D333 found in hydrophilic loops were also found to be critical to Oac function.

**Conclusions:**

By studying the effect of the mutations on Oac’s function and assembly, an insight into the possible roles played by the chosen amino acids in Oac was gained. The transmembrane serine-glycine motifs and hydrophilic residues (RK 110–111, GR 269–270 and D333) were shown to have an affect on Oac assembly which suggests a structural role for these motifs. The phenylalanine-proline and the tryptophan-threonine motifs affect Oac function which could suggest a catalytic role for these amino acids.

## Background

Thirteen serotypes of *S. flexneri* have been recognised, all of which are capable of causing bacillary dysentery or shigellosis. The difference in the various serotypes arises from variations in the nature of their lipopolysaccharide (LPS) O-antigen structure. All serotypes (except serotype 6) share a common O-antigen backbone structure consisting of repeating units of a tetrasaccharide made up of an N-acetylglucosamine residue linked to three rhamnose residues [1]. Glucosyl and/or *O*-acetyl groups added to the backbone allow diversification of the parent serotype into different serotypes. The factor responsible for adding an acetyl group to the Rhamnose 3 molecule on the O-antigen backbone has been identified to be an integral membrane protein known as *O*-acetyltransferase or Oac encoded by the temperate bacteriophage Sf6 [2]. Oac is capable of acetylating the O-antigen of serotypes Y, X, 1a and 4a and changing the antigenic signature of the parent *S. flexneri* strain to generate a new serotype (serotypes 3b, 3a, 1b and 4b, respectively).

We previously reported the solved two-dimensional membrane topology of Oac [3]. Using a gene-fusion approach with dual reporter genes *pho*A-*lac*Zα, Oac’s topology with reference to the cytoplasmic membrane was proposed. Oac consists of ten α-helical membrane spanning regions with both the N and C-termini located in the cytoplasm.

Although knowledge about integral membrane acetyltransferases belonging to *S. flexneri* and other Gram-negative bacteria is mounting, the actual mechanism of serotype conversion mediated by these proteins remains largely unknown. An understanding of the key structural and functional components involved in *O-*acetylation is thus required to give an insight into the overall mechanism. Several studies on acetyltransferases belonging to different organisms have been carried out to identify residues playing critical roles [4-7]. Critical residues have been identified in acetyltransferases in these studies either by the isolation of spontaneous phenotype-modifying mutants or by targeted mutagenesis of residues.

In this study, Oac amino acids which showed a high level of conservation with those of other homologous acetyltransferases were identified by BLASTp searches and subsequent alignment of the protein sequences having the highest hits with *S. flexneri* Oac. This information was coupled with Oac’s topology data to obtain a visual representation of the location of the conserved residues in Oac. The identified conserved residues were then subjected to mutagenesis in order to study their effects on Oac. Based on the findings, roles for the different amino acids in context to their location are proposed.

## Results

### Construction of mutant proteins

**Figure 1:**
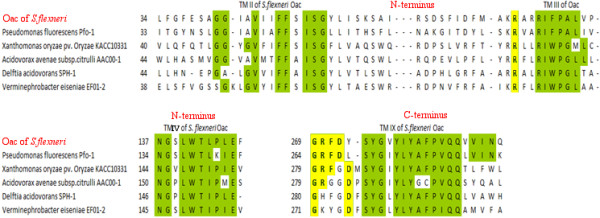
** Alignment of BLASTp hits of* S. flexneri *Oac with homologous acetyltransferases.** Regions highlighted in green are conserved amino acids found within TM segments, while yellow highlighted regions indicate conserved residues in cytoplasmic loops.

Oac and its homologues grouped under protein super-family COG1835 (consisting of predicted acetyltransferases) show high levels of conservation of several amino acid motifs at similar positions of the proteins (Figure 1, Additional File 1: Table AFT1 and [8]). The conservation of these motifs suggests that they may be involved in specific conserved functions. The conservation seems to be almost strictly confined to the amino terminus of the protein of about 160 amino acids, with four distinctly identifiable motifs (GGxxxV, FFxISG, RIxPxL, NGxLWT), all of which are found in the transmembrane segments. Amino acids which are conserved to a lesser extent are also found in close proximity to these motifs. Two more motifs (SYGxY and FPVQQ) are seen in the transmembrane segments in the carboxyl terminus of the proteins. Between Oac and its closest homologue, the Acetyltransferase 3 of *Pseudomonas fluoroscenes*, there are additional conserved motifs (FxPSMMLSA in TM X), suggesting a possible common ancestor and/or common function or mode of action for these two proteins.

**Figure 2:**
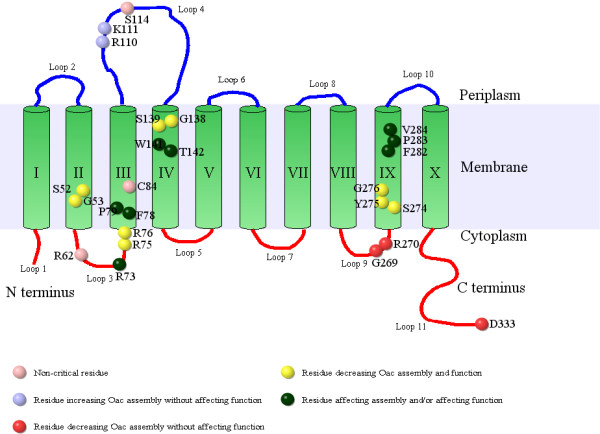
** Amino acid residues of Oac selected for mutagenesis.** The topology model of Oac [3] has been used to show the location of the residues targeted for mutagenesis. Residues have been colour-coded based in their effects on Oac assembly and function as outlined in Table 2. Accordingly, non-critical residues are shown in light pink, residues affecting Oac assembly (increase) without affecting function are shown in blue, residues affecting Oac assembly (decrease) without affecting function are shown in dark pink, residues affecting Oac assembly (decrease) and function are shown in yellow, residues affecting either assembly (increase) and/or function are shown in green.

Site-directed mutagenesis was used to target specific residues in these motifs as outlined in Table 1 and Figure 2. All residues were mutated to the neutral amino acid alanine since its neutral charge and small size is expected to impose minimum electrostatic and stearic hindrance [9]. The residues R 62, R 73, RR 75–76, RK 110–111 and S114 were previously targeted [3]. In this study, we also assessed these residues for quantitative assessment of the assembly of the protein in the membrane.

**Table 1:** Amino acid residues in Oac targeted for mutagenesis

**Mutation**	**Location of mutation in Oac**	**Basis of selection for mutagenesis**
SG 52-53	TM II	Part of conserved motif FFiSG
R 62	Loop 3	Positively charged amino acid in cytoplasmic loop. Shows some level of conservation with homologous acetyltransferases.
R 73	Loop 3	Positively charged amino acid in cytoplasmic loop. Shows some level of conservation with homologous acetyltransferases.
RR 75-76	Loop 3	Positively charged amino acid R75 shows some level of conservation. R76 is highly conserved and belongs to motif RIfPAL.
FP 78-79	TM III	Part of conserved motif RIfPAL.
C 84	TM III	To investigate if it forms disulphide bonds with other cysteine residues in Oac.
RK 110-111	Loop 4	Positively charged amino acid in periplasmic loop. Not conserved in location; thus mutated to determine if amino acid charge was important to position.
S 114	Loop 4	Neutral amino acid in periplasmic loop. Not conserved in location; thus mutated to determine if amino acid charge was important to position.
GS 138-139	TM IV	Part of conserved motif NGsLWT
WT 141-142	TM IV	Part of conserved motif NGsLWT
GR 269-270	Loop 9	Shows some level of conservation with homologous acetyltransferases. Present in a cytoplasmic loop
SYG 274-276	TM IX	Part of conserved motif SYGxY
FPV 282-284	TM IX	Part of conserved motif FPVQQ
D 333	Loop 11	Negatively charged amino acid in cytoplasmic loop. Not conserved in location; thus mutated to determine if amino acid charge was important to position.

The location of residues targeted for mutagenesis and the rationale for their selection have been described. The red colour indicates residues chosen for mutagenesis. Lower case characters in motifs represent residues which are found only in Oac and are not conserved.

### Functional assessment of mutants

**Figure 3:**
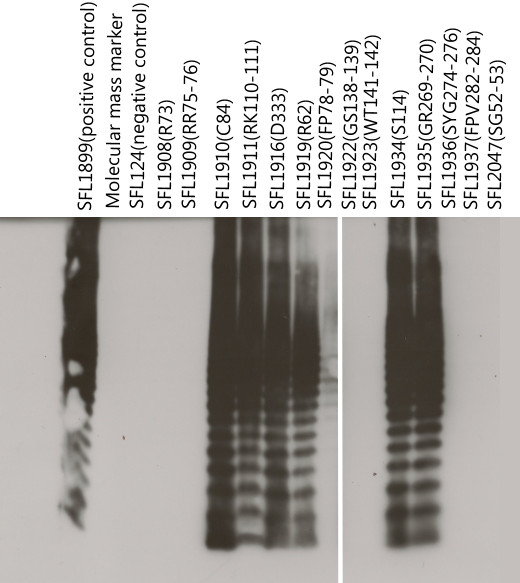
** SDS-PAGE of* S. flexneri *LPS preparations of Oac mutants.** LPS was subjected to Western blotting using MASF6 primary antibodies and anti-mouse IgG-HRP conjugated secondary antibodies. All samples contained approximately equal amount of LPS preparation as judged by preceding silver stained gels (gels not shown). SFL1899 is the positive control and SFL124 is the negative control. The Molecular mass marker used was the PageRuler^TM^ prestained protein ladder (Fermentas).

The mutant constructs obtained were transformed into the *S. flexneri* serotype Y strain (SFL124) to assess Oac functionality by conversion to serotype 3b, the results of which are summarized in Table 2. Slide agglutination was used as a preliminary test to observe the functional expression of Oac followed by LPS Western immunoblotting to confirm the result (Figure 3). Four strains namely SFL1908 (mutant R73), SFL1909 (mutant RR75-76), SFL1936 (mutant SYG 274–276) and SFL1937 (mutant FPV 282–284) did not react to the MASF6 (Monoclonal antibody to *S. flexneri* group 6 epitope) antibodies either by the agglutination or in LPS profiling suggesting that these residues were critical to Oac. LPS profiling, however revealed other residues which abolished the function of Oac which were not discernable by slide agglutination. SFL1922 (mutant GS 138–139), SFL1923 (mutant WT 141–142), SFL2047 (mutant SG 52–53) showed an absence of reaction while SFL1920 (mutant FP 78–79) showed a partial reaction only in the larger O-antigen units (probably as a result of cumulative effect of *O*-acetylation). Two more independent repeats of these experiments confirmed the observation.

**Table 2:** Effect of mutations on Oac assembly and function

*** E. coli/Shigella *strain**	**Mutation**	**Effect of mutation on Oac-PhoA-LacZα assembly in membrane (compared to wild type protein assembly)**		**Effect of mutation on Oac function (compared to wild type protein function)**		**Overall effect on Oac**
		**Western blot analysis**	**β-galactosidase activity in Miller units with standard deviations shown in parentheses**	**Slide agglutination reaction to MASF6**	**LPS reaction to MASF6**	
B1790	_	Not assembled (negative control)	3.8 (0.73)	N/A	N/A	N/A
SFL124	Serotype Y strain	N/A	N/A	_	_	N/A
B2012/SFL1899	Wild type Oac	Assembled (Positive control)	120.19 (5.27)	+	+	Positive control
B2266/SFL2047	SG 52-53	Appears decreased	34.85 (3.11)	+	_	Critical to assembly
B2254/SFL1909	RR 75-76	Appears decreased	79.43 (5.25)	_	_	Critical to assembly
B2260/SFL1922	GS 138-139	Appears decreased	73.87 (9.85)	+	_	Critical to assembly
B2264/SFL1936	SYG 274-276	Appears decreased	40.38 (3.40)	_	_	Critical to assembly
B2256/SFL1916	D 333	Appears decreased	46.25 (11.17)	+	+	Critical to assembly
B2263/SFL1935	GR 269-270	Appears decreased	77.17 (14.53)	+	+	Critical to assembly
B2255/SFL1911	RK 110-111	Appears increased	207.23 (16.77)	+	+	Critical to assembly
B2253/SFL1908	R 73	Appears increased	176.08 (11.77)	_	_	Critical to assembly/function
B2258/SFL1920	FP 78-79	Appears increased	156.45 (4.45)	+	+/_	Critical to assembly/function
B2261/SFL1923	WT 141-142	Appears increased	174.76 (20.79)	+	_	Critical to assembly/function
B2265/SFL1937	FPV 282-284	Appears increased	163.78 (9.49)	_	_	Critical to assembly/function
B2257/SFL1919	R 62	Appears increased	122.05 (13.34)	+	+	Non-critical
B2021/SFL1910	C 84	No effect	124.54 (7.39)	+	+	Non-critical
B2262/SFL1934	S 114	Appears increased	132.32 (10.99)	+	+	Non-critical

The table compares the overall effects of the various mutations on Oac assembly and function. Both qualitative and quantitative assays were considered to represent the effect on the protein assembly. Firstly, Western immunoblotting was used as a qualitative assay to assess the assembly of wild type and mutant Oac-PhoA-LacZα proteins. Levels of Oac- PhoA-LacZα protein assembled by the mutant constructs were then quantified and compared with the wild type protein using the BG (β-galactosidase) assay. Results are an average of two independent experimental repeats with two internal replicates. The table also shows the results of the qualitative functional assays conducted for the Oac mutants. Preliminary slide agglutination tests were confirmed by LPS Western immunoblotting using MASF6 antibodies. N/A refers to not applicable.

### Assessment of the role of the residues in protein assembly in the membrane–qualitative analysis

**Figure 4:**
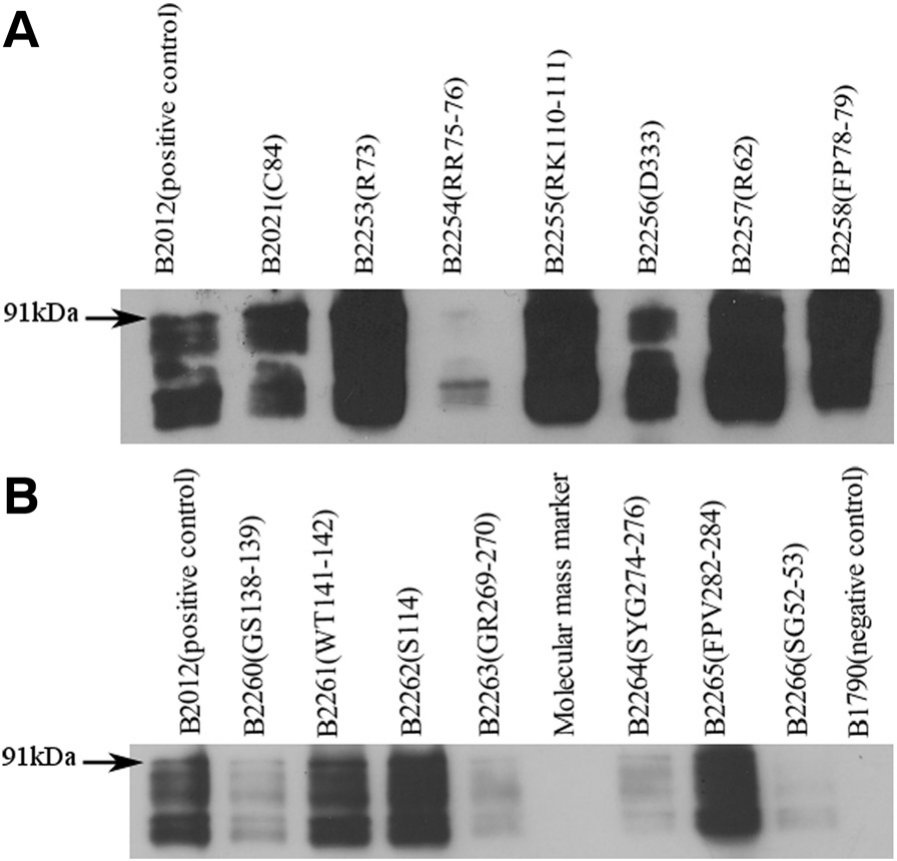
** A and B: SDS-PAGE of membrane protein of Oac mutants.** Proteins were subjected to Western immunoblotting using anti-PhoA antisera primary antibodies and anti-mouse IgG HRP conjugated secondary antibodies. Approximately 10 μg of protein was loaded in a 10% SDS-PAGE gel. B2012 is the positive control, B1790 is the negative control. The Molecular mass marker used was the PageRuler^TM^ prestained protein ladder (Fermentas). The 91 kDa Oac-PhoA-LacZα protein band is indicated.

To determine whether or not the introduced mutations affected protein assembly in the membrane and hence function, all mutant proteins were constructed with the reporter protein PhoA-LacZα which provided a means of assessing membrane protein assembly using anti-PhoA antibodies in Western blotting (Figure 4a and b). Proteins with mutations R62, R73, FP 78–79, C84, RK110-111, S114, WT 141–142, and FPV 282–284 all appear to be synthesised and assembled in the membrane as seen by the ~91 kDa Oac-PhoA-LacZα fusion protein band. Some degradation of the fusion protein was observed as additional lower molecular weight signals were seen. Relative differences in the assembly of the mutated proteins compared with the non-mutated Oac-PhoA-LacZα fusion protein seem to exist and further analyses of these differences are outlined below. Mutations SG 52–53, RR75-76, GS 138–139, GR 269–270, SYG 274–276 and D333 all caused reduced protein assembly. The qualitative assessment of their assembly on the dual indicator (DI) plate indicated (data not shown) the proteins synthesised by these mutant strains were assembled in the membrane (as indicated by red colony coloration seen for these strains similar to the non-mutated strain B2012). Western blot analysis of the mutants indicates the degree of assembly and the comparison of similar amounts of protein revealed differences in the extent of assembly.

### Quantitative assessment of the extent of protein assembly in the membrane

Differences in the levels of Oac- PhoA-LacZα protein assembled by the mutant constructs were quantified and compared with the wild type using the BG (β-galactosidase) assay (Table 2). Overall, the BG values obtained correlate well with the observations made by the membrane protein Western blotting. Mutations R62, C84 and S114 had BG values similar to that seen for B2012 (harbouring pNV1644). The high BG values obtained for mutations R73, FP 78–79, RK110-111, WT 141–142 and FPV 282–284, are consistent with the high level of expression observed in the membrane protein Western blots. Those proteins which displayed reduced levels of assembly based on the Western blot, SG 52–53, RR 75–76, GS 138–139, GR269-270, SYG 274–276 and D333, correspond with reduced BG activities.

Table 2 summarises the results obtained from both the qualitative and quantitative assays performed to assess the role played by the mutants in Oac’s functioning and assembly in the membrane.

## Discussion

This study, aimed at investigating amino acid residues critical to Oac, forms an important milestone towards understanding the mechanistics of acetyltransferases in general. An alignment of proteins homologous to Oac revealed several conserved amino acids mainly present in the TM helical regions. Accordingly, these residues were targeted for mutagenesis. All residues mutated were then assessed for their effect on Oac’s function and assembly in the cytoplasmic membrane. In order to assign specific roles for all the other critical amino acids identified, the effects of the mutations on protein assembly in the membrane has been used as an initial criteria followed by the effect on function, since naturally under *in vivo* conditions, protein (synthesis and) assembly pre-determine functional capabilities.

From these studies three non-essential amino acids of Oac have been identified. Amino acids R62, C84 and S114 could be replaced without having any adverse effects on either the function or the assembly of Oac. Among the three amino acids only the location of R62 appears fairly conserved (in four of the seven homologous proteins used for the alignment); however this positional conservation does not appear to offer any importance to Oac.

Arginine residues have been frequently implicated to play a structural role [5,6] and also to function as active-site residues in acetyltransferases of different organisms [10-12]. We previously reported the identification of critical arginines (R73 and RR 75–76) [3]. In this study the inclusion of the quantitative assessment of protein assembly has provided a better insight into the role played by these residues. It appears that although these residues are in close proximity to one another, they perform different roles in Oac. RR 75–76 appear to disrupt the assembly of the protein suggesting they have a structural role, while the absence of function in R73 despite protein assembly (as seen in [3]) suggests it either causes the assembly of an inefficient form of the protein or it directly relates to the specific absence of catalytic residue.

The replacement of the two basic residues, RK110-111, with alanines increases protein assembly and function. The specific location of this residue pair could be a contributing factor to the very different role these residues play. On the basis of the activities observed in this study, it could be hypothesised that the RK 110–111 motif assists in regulating the kinetics of protein assembly and folding at an optimal rate (and quantity) suited for the overall purpose of Oac in *O*-acetylation. Similar effects were shown by Loladze *et al. *[13] with ubiquitin, where the substitution of basic amino acids Lys, Arg and His, with both neutral and acidic amino acids increased overall protein stability.

The definite role of the GR 269–270 and D333 residues is not clear. Although not critical to function, the drastic impact on their assembly indicates that the localized charges of the amino acids may have effects which are too subtle to be picked up by phenotype evaluations. The effect caused by GR 269–270 could be due to the elimination of the positive charge brought by the arginine residue which might be required to maintain the short loop 9 in the cytoplasm. Moreover, glycine residues possess considerable conformational flexibility which might also be necessary to maintain loop structures. The elimination of the glycine residue might have, if not drastically, interfered with the assembly of the protein beyond the amino acid position 269.

There is an abundance of glycine and serine residues, either as a pair or in close proximity to one other in most of the helices of Oac, but the motifs targeted for mutagenesis (SG 52–53, GS 138–139 and SYG 274–276) show a high level of conservation among other homologous acetyltransferases. Luck *et al. *[4] reported the occurrence of a spontaneous mutation in the *lag*-1 gene which encodes an O-acetyltransferase in *Legionella pneumophilia* resulting in the loss of enzyme activity without affecting mRNA transcription levels. An analysis of the DNA sequence revealed that a single nucleotide substitution resulted in the serine residue of the FFWLSG motif being mutated to leucine. This motif corresponds to Oac’s FFxISG motif containing SG 52–53 and in the current study it has been established that SG 52–53 and the other SG motifs affect protein assembly, although levels of transcription have not been investigated. All three of Oac’s SG motifs appear to be important to maintain the structural integrity of Oac. Similar opinions on the importance of glycine and serine residues occurring together and providing folding stability to membrane proteins have been expressed by other research groups [14,15].

The conserved motifs, FP 78–79 and FPV 282–84, are found in transmembrane segments III and IX, respectively. Alanine replacements of these residues affected Oac function in spite of the fact that the protein assembly appeared to be more than what was seen for the wild type Oac. The effects on assembly could partly be explained by the absence of proline residues. Proline residues are normally not favoured in α-helices because they are thought to produce ‘kinks’ in transmembrane helices, thereby introducing destabilising energy on the helix [16]. The loss of a proline could have thus provided additional stability to the protein thereby increasing its assembly. The effect on function could either suggest that a conformationally unstable protein was assembled or that it truly represented the specific absence of a catalytic residue. It is possible that the conserved proline residue had a far more critical purpose relating to function. A functional role for prolines has been shown in another class of enzymes – the phenylalanine-specific permease of *E. coli *[17]. The conserved phenylalanine or valine (in FPV) residues could also have an effect on function. With phenylalanine, continuing from the identification of critical Arg9 and Arg64 (by Delomenie *et al. *[6] in human arylamine N-acetyltransferases NAT1 and NAT2), Goodfellow *et al. *[7] identified a critical transmembrane phenylalanine in these enzymes. A Phe125 was found to be involved in determining substrate selectivity leading to the hypothesis that like the arginines, the phenylalanine residue might form part of the active site. A similar arrangement of critical residues appears to exist in Oac. Critical R73, RR 75–76 lie in close proximity to FP 78–79 and all may be involved in forming one of the active sites.

Like the phenylalanine and proline motifs described above, the tryptophan-threonine pair (WT141-142) in TM IV appears to be important to Oac’s function. The effect of these residues in acetyltransferases is largely unknown, although tryptophan residues have been found to be critical in membrane proteins belonging to transporter families [18,19]. In this study, tryptophan was replaced with alanine. Both are neutrally charged residues, but due to the presence of two fused aromatic rings, tryptophan is a considerably larger molecule than alanine. If tryptophan was involved in some aspect of catalysis, like perhaps the interaction with another residue bearing the captured acetyl group, it is possible that the extra spatial volume it provides (compared with alanine) is necessary to make the two residues interact.

## Conclusion

The conserved and non-conserved Oac amino acids mutated in this study were assessed for their effect on Oac’s function and assembly. Conserved arginine residues such as R73, RR75-76, serine-glycine motifs (SG 52–53, GS 138–139 and SYG 274–276), phenylalanine-proline motifs (FP 78–79 and FPV 282–84) and motifs WT141-142 and GR269-270 were found to be critical to Oac. Non-conserved RK110-111 and D333 were also found to be critical. While the identification of conserved essential amino acids could suggest a common requirement among other homologous acetyltransferase, the identification of the non-conserved but critical amino acids found uniquely in *S. flexneri* Oac suggests they play a specialised role. Based on their effects on Oac function and assembly, an insight into the possible roles of these residues has been gained. This could form the basis of future studies to understand the overall mechanism of action of Oac.

## Methods

### Bacterial strains and culture conditions

*E. coli* strain JM109 [20] was used as a host strain for propagating all plasmids used in this study. Functional expression of plasmid constructs was tested using *S. flexneri* serotype Y strain (SFL 124) which is an attenuated vaccine candidate strain [21]. Details of the strains created are described in Additional File 1: Table AFT2 and Additional file 1: Table AFT3. All cultures were grown at 37°C aerobically in Luria-Bertani media with chloramphenicol (Cm-25 *μ*g/ml final concentration).

### DNA techniques

Plasmid vectors were derived from pBCSK + (Stratagene). Plasmids were isolated using the alkaline lysis method either using the QIAprep spin miniprep kit (Qiagen) or by the method described by Sambrook *et al. *[20]. Details of the plasmid constructs made in this study are described in Additional File 1: Table AFT2. Standard procedures as outlined in Sambrook *et al. *[22] were used for cloning and transformation by electroporation.

Sequencing was performed at the Biomolecular Resource Facility, John Curtin School of Medical Research, Australian National University, using the Big Dye Terminator v3.1 Cycle Sequencing Kit.

### Creation of *oac*-*pho*A-*lac*Zα fusion construct pNV1644

The construction of the plasmid pNV1644 (in B2012) and SFL1899 has previously been described in [3]. When B2012 (carrying pNV1644) was plated on DI media, a red coloured fusion product was observed indicating that the point of fusion, that is the C-terminal end, was localized in the cytoplasm. Furthermore, as described in [3], SFL1899 containing pNV1644 was confirmed by slide agglutination and LPS Western immunoblotting using MASF 6 antibodies to carry a functional *oac* gene.

pNV1644 was used as a parent plasmid to make constructs containing *oac* point mutations. All subsequent constructs generated could be assessed against the parent strain for Oac functionality and assembly in the membrane. For instance, the observation of colonies with red colouration similar to that seen for pNV1644 (B2012) would indicate that the mutated protein was fully and correctly assembled similar to that of the wild-type Oac. Moreover, with the use of anti-PhoA antibodies in Western immunoblotting, the level of expression of the modified Oac-PhoA-LacZα fusion proteins could be assessed and compared with the unmodified Oac-PhoA-LacZα fusion protein.

### Oac functionality testing

*O*-acetylation establishes the group 6 specificity of the O-antigen. This was tested by transforming a serotype Y strain, SFL124 with the appropriate construct encoding Oac and observing whether the serotype Y strain (group specificity 3, 4) is converted to serotype 3b, having group specificities of 6 and 3, 4. All tests were done in parallel with SFL1899 (having functional *oac* in pNV1644) serving as a positive control and SFL124 serving as negative control for Oac functionality.

Slide agglutination was performed using antisera (Denka-Seiken, Tokyo) or monoclonal antibodies such as MASF6 (Reagensia AB, Sweden). Absence of autoagglutination was always confirmed using saline instead of the antibodies. All samples were subjected to LPS Western immunoblotting as a confirmatory test after the preliminary result obtained from the slide agglutination. All functional results are thus shown for both the slide agglutination and the LPS Western blotting.

Western blots for LPS samples were prepared and analysed using 12% resolving gels and silver-staining as described by Hitchcock and Brown [23]. Silver staining helped ensure equal loading of samples. The same volume of sample was then used to run another gel for the Western blotting using the MASF 6 as the primary antibody (diluted 1 in 200) and anti-mouse IgG HRP-conjugated as the secondary antibody (diluted 1 in 8000). Detection was performed using the Super Sugnal West Pico Chemiluminescent Substrate according to the manufacturer’s directions (Pierce).

### Assessment of the role of the residues in protein assembly in the membrane - qualitative analysis

Membrane fractions of all mutant proteins were prepared as described by Morona *et al. *[24] and used in Western immunoblotting. Prior to loading samples on SDS-PAGE, the total amount of protein present in the samples was estimated by the Pierce bicinchoninic acid (BCA) Protein Assay Kit (Thermo Scientific). Approximately 10 μg of protein was loaded in a 10% SDS-PAGE gel. Western blots were carried out using anti-PhoA antisera (Chemicon) in a 1 in 1000 dilution as the primary antibody and anti-mouse IgG-HRP conjugated secondary antibody (Sigma) in a 1 in 8000 dilution as the secondary antibody. Detection was performed as described above.

### Quantitative assessment of the extent of protein assembly in the membrane- BG assay

pNV1644 carries the *pho*A-*lac*Zα reporter gene and hence codes for functional LacZα (otherwise BG). All mutant constructs were assessed against pNV1644 to determine whether the mutation affected the assembly of the Oac-PhoA-LacZα protein and hence altered the BG activity. A quantitative assay was used to measure BG activities based on the cleavage of the substrate *o-*nitrophenyl- β-D-galactoside as described by Miller *et al. *[25]. Before the BG assay was performed, strain B2280 (containing pNV1870) was generated from pNV1644. pNV1870 was constructed to have the dual reporter fused immediately after the *oac* promoter with the rest of *oac* deleted. In short, the dual reporter would be transcribed under the influence of the *oac* promoter in the same background vector pNV1644 used to make all the mutant constructs. The rationale behind constructing this vector was that it was necessary to see what levels of BG activity were obtained for the LacZα fragment alone, which was fully assembled and active in the cytoplasm without being dependent on the assembly and folding of the Oac membrane protein. Thus pNV1870 was constructed using pNV1644 as the template DNA using a reverse PCR technique (as described for pNV1644 in [3]). Primers Pholac(BamHI)_For and Oac_K3(BamHI)_Rev (Additional File 1: Table AFT4) which bound downstream and upstream, respectively, of the *oac* sequence, amplified the entire vector excluding the *oac* coding sequence.

Negative controls were also included in the assay to monitor leaky expression of BG. Baseline BG activities recorded were then subtracted from sample values. Values obtained are an average of two independent experimental repeats with internal replicates of the samples.

### Site directed mutagenesis

Mutagenesis was performed using the Stratagene’s QuickChange Site-Directed Mutagenesis protocol. Details of the primers used for site-directed mutagenesis are presented in Additional File 1: Table AFT4. All constructs were sequenced entirely using appropriate primers to ensure that the intended mutation was in place and that no other extraneous mutations had been introduced during the PCR process.

## Abbreviations

BCA, Bicinchoninic acid; BG, β- galactosidase; DI, Dual indicator; HRP, Horse radish peroxidise; LPS, Lipopolysaccharide; MASF, Monoclonal antibody for Shigella flexneri; NAT, N-acetyltransferase; Oac, O-acetyltransferase; TM, Transmembrane.

## Competing interests

The authors declare that they have no competing interests.

## Authors’ contributions

FT contributed to the experimental design of the study, carried out all experiments and analyses and drafted the manuscript. NKV contributed to the experimental design and supervised the study, analysed results and critically revised the manuscript. Both authors have read and approved the final manuscript.

## Supplementary Material

Additional file 1 **Table AFT1:** Proteins chosen for alignment with Sf6 Oac (as shown in Figure 1). **Table AFT2: ***E*. *coli* strains used and created in this study with details of the plasmids they contain. **Table AFT3: ***S. flexneri* strains used and created in this study. **Table AFT4:** Oligonucleotide primers used in this study. Click here for file
