# Supplementary material for: Identification of critical residues of the serotype modifying O-acetyltransferase of Shigella flexneri
Source: BMC Biochem. 2012 Jul 15;13:13. doi: 10.1186/1471-2091-13-13 (PMC3467182; doi:10.1186/1471-2091-13-13)
Supplement: Additional file 1 — Table AFT1: Proteins chosen for alignment with Sf6 Oac (as shown in Figure 1). Table AFT2: E. coli strains used and created in this study with details of the plasmids they contain. Table AFT3: S. flexneri strains used and created in this study. Table AFT4: Oligonucleotide primers used in this study. [file 1471-2091-13-13-S1.docx]

**Table AFT1:** Proteins chosen for alignment with Sf6 Oac (as shown in Figure 1)

| **Protein name** | **Source** | **Length of protein** | **Reference numbers** |
| --- | --- | --- | --- |
| *O*-acetyl transferase | Enterobacteria phage Sf6 | 333 aa | Embl accession X56800.1 |
| Acyltransferase 3 | Pseudomonas fluorescens Pf0-1 | 336 aa | NCBI Reference Sequence: YP_350922.1 |
| *O*-antigen acetylase | Xanthomonas oryzae pv. oryzae KACC 10331 | 350 aa | NCBI Reference Sequence: YP_199421.1 |
| Putative O-antigen acetylase | Acidovorax citrulli AAC00-1 | 351 aa | NCBI Reference Sequence: YP_972376.1 |
| Acyltransferase 3 | Delftia acidovorans SPH-1 | 358 aa | NCBI Reference Sequence: YP_001561884.1 |
| Acyltransferase 3 | Verminephrobacter eiseniae EF01-2 | 339 aa | NCBI Reference Sequence: YP_996959.1 |

**Table AFT2:** *E*. *coli* strains used and created in this study with details of the plasmids they contain.

| **Strain** | **Description** | **Characteristics** | **Source** |
| --- | --- | --- | --- |
| JM109 | Host strain | *rec*A1 *sup*E44 *end*A1 *hsd*R17 *gyr*A96  *rel*A1 *thi* ∆(*lac*-*pro*AB) [F' *tra*D36 *pro*AB *lac*Iq *lac*Z∆M15] | [18] |
| B1790 | JM109 carrying pNV1492 | 1.0 kb *oac* fragment ligated between *Nhe*I and *Bam*HI sites of pNV1473. *oac* out-of- frame with *pho*A-*lac*Zα | Lab strain |
| B2012 | JM109 carrying pNV1644 | pNV1492 used as template to fuse full length *oac* to *pho*A-*lac*Zα with *Stu*I at fusion point.*oac* in-frame with *pho*A-*lac*Zα | This study |
| B2021 | JM109 carrying pNV1653 | pNV1644 used as template to mutate sequence corresponding to C 84 to alanine | This study |
| B2253 | JM109 carrying pNV1651 | pNV1644 used as template to mutate sequence corresponding to R 73 to alanine | This study |
| B2254 | JM109 carrying pNV1652 | pNV1644 used as template to mutate sequence corresponding to RR 75-76 to alanines | This study |
| B2255 | JM109 carrying pNV1654 | pNV1644 used as template to mutate sequence corresponding to RK 110-111 to alanines | This study |
| B2256 | JM109 carrying pNV1670 | pNV1644 used as template to mutate sequence corresponding to D 333 to alanine | This study |
| B2257 | JM109 carrying pNV1672 | pNV1644 used as template to mutate sequence corresponding to R 62 to alanine | This study |
| B2258 | JM109 carrying pNV1680 | pNV1644 used as template to mutate sequence corresponding to FP 78-79 to alanines | This study |
| B2260 | JM109 carrying pNV1682 | pNV1644 used as template to mutate sequence corresponding to GS 138-139 to alanines | This study |
| B2261 | JM109 carrying pNV1683 | pNV1644 used as template to mutate sequence corresponding to WT 141-142 to alanines | This study |
| B2262 | JM109 carrying pNV1716 | pNV1644 used as template to mutate sequence corresponding to S 114 to alanine | This study |
| B2263 | JM109 carrying pNV1717 | pNV1644 used as template to mutate sequence corresponding to GR 269-270 to alanines | This study |
| B2264 | JM109 carrying pNV1718 | pNV1644 used as template to mutate sequence corresponding to SYG 274-276 to alanines | This study |
| B2265 | JM109 carrying pNV1719 | pNV1644 used as template to mutate sequence corresponding to FPV 282-284 to alanines | This study |
| B2266 | JM109 carrying pNV1822 | pNV1644 used as template to mutate sequence corresponding to SG 52-53 to alanines | This study |
| B2280 | JM109 carrying pNV1870 | pNV1644 used as template to fuse *oac* bp 9 to *pho*A-*lac*Zα start with *Bam*HI at fusion point | This study |

**Table AFT3:** *S. flexneri* strains used and created in this study

| **Strain** | **Characteristics** | **Source** |
| --- | --- | --- |
| SFL124 | Attenuated serotype Y vaccine candidate (∆*aro*D) | [19] |
| SFL1899 | SFL124 carrying pNV1644 | This study |
| SFL1908 | SFL124 carrying pNV1651 | This study |
| SFL1909 | SFL124 carrying pNV1652 | This study |
| SFL1910 | SFL124 carrying pNV1653 | This study |
| SFL1911 | SFL124 carrying pNV1654 | This study |
| SFL1916 | SFL124 carrying pNV1670 | This study |
| SFL1919 | SFL124 carrying pNV1672 | This study |
| SFL1920 | SFL124 carrying pNV1680 | This study |
| SFL1922 | SFL124 carrying pNV1682 | This study |
| SFL1923 | SFL124 carrying pNV1683 | This study |
| SFL1934 | SFL124 carrying pNV1716 | This study |
| SFL1935 | SFL124 carrying pNV1717 | This study |
| SFL1936 | SFL124 carrying pNV1718 | This study |
| SFL1937 | SFL124 carrying pNV1719 | This study |
| SFL2047 | SFL124 carrying pNV1822 | This study |

**Table AFT4:** Oligonucleotide primers used in this study

| **Primer name** | **Sequence (5’ to 3’)** | **Binding site/purpose** |
| --- | --- | --- |
| Pholac(BamHI)_For | CGCGGATCCGTTCTGGAAAACCGGGCTGC | Binds downstream of *oac* in pNV1644 |
| Oac_K3(BamHI)_Rev | CGCGGATCCCTTATGCATTAAAATAAAGCCTCTAAG | Binds upstream of *oac* in pNV1644 |
| FT_Oac-SG52,53_For | CATATTCTTTTCAATAGCAGCATATCTGATTTC | To mutate residues SG 52-53 of Oac to alanines |
| FT-Oac-SG52,53-Rev | GAAATCAGATATGCTGCTATTGAAAAGAATATG | To mutate residues SG 52-53 of Oac to alanines |
| FT_Oac_R62A_For | CAAAATCAGCTATCGCGAGTGATTCCTTTATTG | To mutate residues R 62 of Oac to alanines |
| FT_Oac_R62A_Rev | CAATAAAGGAATCACTCGCGATAGCTGATTTTG | To mutate residues R 62 of Oac to alanines |
| Oac_R73A_For | GATTTCATGGCTAAAGCAGCGAGAAGAATATTCC | To mutate residues R 73 of Oac to alanine |
| Oac_R73A_Rev | GGAATATTCTTCTCGCTGCTTTAGCCATGAAATC | To mutate residues R 73 of Oac to alanine |
| Oac_R75A_R76A_For | CATGGCTAAAAGAGCGGCAGCAATATTCCCGGCG | To mutate residues RR 75-76 of Oac to alanines |
| Oac_R75A_R76A_Rev | CGCCGGGAATATTGCTGCCGCTCTTTTAGCCATG | To mutate residues RR 75-76 of Oac to alanines |
| FT_Oac-FP78,79_For | CGAGAAGAATAGCCGCGGCGCTGGTCC | To mutate residues FP 78-79 of Oac to alanines |
| FT_Oac-FP78,79_Rev | GGACCAGCGCCGCGGCTATTCTTCTCG | To mutate residues FP 78-79 of Oac to alanines |
| Oac_C84A_For | GCTGGTCCCTGCCTCTATACTGAC | To mutate residues C 84 of Oac to alanine |
| Oac_C84A_Rev | GTCAGTATAGAGGCAGGGACCAGC | To mutate residues C 84 of Oac to alanine |
| Oac_R110A_K111A_For | CCATGACATTGTCGCGGCGACCATAAGCTC | To mutate residues RK 110-111 of Oac to alanines |
| Oac_R110A_K111A_Rev | GAGCTTATGGTCGCCGCGACAATGTCATGG | To mutate residues RK 110-111 of Oac to alanines |
| FT_Oac-S114A_For | GGAAGACCATAGCCTCTATTTTTATG | To mutate residues S114 of Oac to alanine |
| FT_Oac-S114A_Rev | CATAAAAATAGAGGCTATGGTCTTCC | To mutate residues S114 of Oac to alanine |
| FT_Oac-GS137,138A_For | GGAATTAACGCCGCTTTGTGGACC | To mutate residues GS 137-138 of Oac to alanines |
| FT_Oac-GS137,138A_Rev | GGTCCACAAAGCGGCGTTAATTCC | To mutate residues GS 137-138 of Oac to alanines |
| FT_Oac-WT140,141A_For | CGGCAGTTTGGCGGCCCTACCGCTTG | To mutate residues WT 140-141 of Oac to alanines |
| FT_Oac-WT140,141A_Rev | CAAGCGGTAGGGCCGCCAAACTGCCG | To mutate residues WT 140-141 of Oac to alanines |
| Oac_GR269_For | CCACTGGTTAAGGCCGCGTTTGATTACTCGTATGG | To mutate residues GR 269-270 of Oac to alanines |
| Oac_GR269_Rev | CCATACGAGTAATCAAACGCGGCCTTAACCAGTGG | To mutate residues GR 269-270 of Oac to alanines |
| Oac_SYG274A_For | GGTTAAGGGCAGGTTTGATTACGCGGCTGCTGTGTACATTTATGCATTCC | To mutate residues SYG 274-276 of Oac to alanines |
| Oac_SYG274A_Rev | GGAATGCATAAATGTACACAGCAGCCGCGTAATCAAACCTGCCCTTAACC | To mutate residues SYG 274-276 of Oac to alanines |
| Oac_FPV282_For | GGTGTGTACATTTATGCAGCCGCGGCTCAGCAGGTTGTTATAAACACATTGC | To mutate residues FPV 282-284 of Oac to alanines |
| Oac_FPV282_Rev | GCAATGTGTTTATAACAACCTGCTGAGCCGCGGCTGCATAAATGTACACACC | To mutate residues FPV 282-284 of Oac to alanines |
| Oac_D333A_For | CCTAAATTATCCCTGGCTAGGCCTGTTCTGG | To mutate residues D333 of Oac to alanine |
| Oac_D333A_Rev | CCAGAACAGGCCTAGCCAGGGATAATTTAGG | To mutate residues D333 of Oac to alanine |
